# *Escherichia coli* FabG 3-ketoacyl-ACP reductase proteins lacking the assigned catalytic triad residues are active enzymes

**DOI:** 10.1016/j.jbc.2021.100365

**Published:** 2021-02-03

**Authors:** Zhe Hu, Jincheng Ma, Yicai Chen, Wenhua Tong, Lei Zhu, Haihong Wang, John E. Cronan

**Affiliations:** 1 Guangdong Provincial Key Laboratory of Protein Function and Regulation in Agricultural Organisms, College of Life Sciences, South China Agricultural University, Guangzhou, Guangdong, China; 2 Department of Microbiology, University of Illinois at Urbana-Champaign, Urbana, Illinois, USA; 3 Department of Biochemistry, University of Illinois at Urbana-Champaign, Urbana, Illinois, USA

**Keywords:** short-chain alcohol dehydrogenase/reductase, 3-ketoacyl-ACP reductase, *E. coli* FabG, fatty acid synthesis, ACP, acyl carrier protein, FAS, fatty acid synthase, SDR, short-chain alcohol dehydrogenase/reductase

## Abstract

The FabG 3-ketoacyl-acyl carrier protein (ACP) reductase of *Escherichia coli* has long been thought to be a classical member of the short-chain alcohol dehydrogenase/reductase (SDR) family. FabG catalyzes the essential 3-ketoacyl-ACP reduction step in the FAS II fatty acid synthesis pathway. Site-directed mutagenesis studies of several other SDR enzymes has identified three highly conserved amino acid residues, Ser, Tyr, and Lys, as the catalytic triad. Structural analyses of *E. coli* FabG suggested the triad S138-Y151-K155 to form a catalytically competent active site. To test this hypothesis, we constructed a series of *E. coli* FabG mutants and tested their 3-ketoacyl-ACP reductase activities both *in vivo* and *in vitro*. Our data show that plasmid-borne FabG mutants, including the double and triple mutants, restored growth of *E. coli* and *Salmonella enterica fabG* temperature-sensitive mutant strains under nonpermissive conditions. *In vitro* assays demonstrated that all of the purified FabG mutant proteins maintained fatty acid synthetic ability, although the activities of the single mutant proteins were 20% to 50% lower than that of wildtype FabG. The S138A, Y151F, and K155A residue substitutions were confirmed by tandem mass spectral sequencing of peptides that spanned all three residues. We conclude that FabG is not a classical short-chain alcohol dehydrogenase/reductase, suggesting that an alternative mode of 3-ketoacyl-ACP reduction awaits discovery.

The architecture of fatty acid synthase (FAS) has three forms (1, 2, 3). Mammalian fatty acid synthase (FAS I) consists of two copies of a single large multifunctional polypeptide derived from a single gene. This structure contains all the active sites and performs all the steps in the synthetic pathway, whereas in yeast and mycobacteria the FAS I enzymes are encoded as two different polyfunctional proteins. Type II bacterial fatty acid synthases (FAS II) are composed of multiple separate enzymes, with each enzyme encoded as a separate open reading frame. Each enzyme catalyzes a discrete step in the pathway. These fundamental differences between the FAS types aids the development of antibacterial agents, although, as yet, none have been of wide clinical utility (1, 4, 5, 6).

The bacterial 3-ketoacyl-ACP reductase (FabG) catalyzes the essential keto reduction step in the elongation cycle of FAS II (1, 2) (Fig. 1*A*). This enzyme is the only known isozyme of this type in bacteria and is highly conserved and ubiquitously expressed in bacteria (2, 7, 8). Thus, FabG has become the focus of numerous attempts to develop antimicrobials (4, 6, 9, 10). Based on its sequence and structure FabG has long been considered a canonical member of a very large family of enzymes, the short-chain alcohol dehydrogenase/reductase (SDR) family, enzymes that perform a wide variety of reduction and dehydrogenation reactions adding or removing hydrogen in a NAD(H)- or NADP-(H)–dependent manner from specific substrates (1, 2, 8). The SDR proteins are generally proteins of 25 to 35 kDa that function as dimers or tetramers (11, 12, 13). Although only 15% to 30% sequence identity exists among different SDR proteins, the SDRs share select distinct sequence motifs such as the N-terminal coenzyme binding site Gly-Xaa_3_-Gly-Xaa-Gly and the centrally located catalytic site Tyr-Xaa_3_-Lys. Mutagenesis of other SDRs has identified Tyr, Lys, and Ser as the catalytic triad residues. The Tyr side chain functions as the catalytic base, the Ser side chain stabilizes the substrate, and the Lys side chain interacts with the nicotinamide ribose and lowers the pKa of the Tyr-OH (11, 12). Note that SDRs have been divided into five types: classical, extended, intermediate, divergent, and complex by Oppermann, Jornvall, and colleagues (11, 12, 13). The most recent review is 12 years old, and, given the rapid expansion of the databases, there may be additional types. Hence, SDRs are rather a hazy grouping. Several sequence signature motifs, most of which are parts of the Rossmann fold, can be identified (Fig. 1) (11, 12, 13).

**Figure 1:**
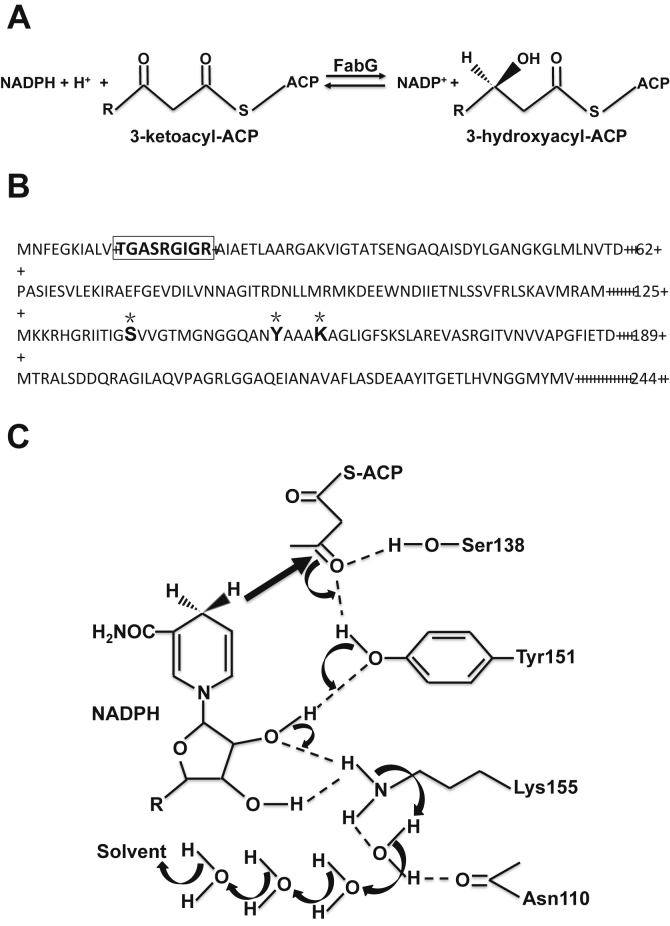
**The 3-ketoacyl-ACP reductase reaction, the amino acid sequence of *E. coli* FabG, and the postulated catalytic mechanism of FabG.***A*, the NADPH-dependent reduction of 3-ketoacyl-ACP catalyzed by 3-ketoacyl-ACP reductase. *B*, the amino acid sequence of *E. coli* FabG. The cofactor binding sequence (Gly motif [GlyXXXGlyXGly]) is boxed. *Asterisks* highlight the putative catalytic triad (S138, Y151, and K155). *C*, The putative FabG catalytic mechanism (15). ACP, acyl carrier protein.

Structural studies of *Escherichia coli* FabG suggested it to be a typical example of an SDR protein. This enzyme of 244 amino acid residues is tetrameric and has an α/β structure with the signature Rossmann fold motif (2, 8, 14, 15, 16, 17). FabG also contains the highly conserved Y151-Xaa3-K155 motif, near the carboxy terminus of helix α4 (Fig. 1*B*). In addition, FabG has a conserved serine residue (S138) located near the tyrosine and lysine residues. Price and colleagues (8, 15) postulated that *E. coli* FabG has a catalytic mechanism similar to that of other SDR enzymes, although they observed that cofactor binding caused a significant conformational change in FabG that buried the signature lysine residue (K155). They proposed that, in *E. coli* FabG, protons are shuttled *via* a water network positioned to serve as a proton wire to the tyrosine *via* K155 and the 2’-hydroxyl of the nicotinamide ribose (8, 15) (Fig. 1*C*). However, in prior work on *Ralstonia solanacearum* FabG1 we substituted Ser143, Y151, and Lys156 with A, F, or A, respectively. To our surprise, these substitutions failed to block the ability of FabG1 to complement growth of an *E. coli fabG* temperature mutant strain at the nonpermissive temperature (Fig. S1). The *R. solanacearum* FabG1 aligns well with *E. coli* FabG (64% identical residues), and the catalytic triad residues (and their spacings) of the two FabG proteins are conserved (Fig. S1). We turned to *E. coli* FabG because it is considered a classical SDR protein and is the subject of several crystal structure studies and genetic analyses. The *R. solanacearum* results suggested that the postulated *E. coli* FabG catalytic triad may not be active site residues. We report that substitution of the putative *E. coli* FabG residues S138, Y151, and K155 with A, F, or A resulted in proteins that retained FabG activity both *in vivo* and *in vitro*.

## Results

### Analysis of the FabG mutants *in vivo*

To test whether the S138-Y151-K155 motif of *E. coli* FabG is the catalytic triad of the enzyme, panels of *E. coli fabG* mutants were constructed on plasmids of high copy and low copy numbers. The plasmids were tested for complementation using two temperature-sensitive mutant strains that lack 3-ketoacyl-ACP reductase activity at 42 °C. We first used *E. coli* strain CL104 and subsequently switched to *Salmonella enterica* serovar Typhimurium LT2 strain CL95 (7). (The use of conditionally mutant strains is required since FabG is an essential enzyme in *E. coli*.) We first substituted FabG S138 with A or T, Y151 with F or H, and K155 with T or A and inserted these mutant *fabG* genes into the high-copy-number vector pBAD24M to produce six plasmids (pTWH22, 23, 24, 25, 29, and 30). Transcription of the genes proceeded from the arabinose-regulated *araBAD* promoter in these plasmids. The vector also supplied the ribosome-binding site necessary for translational initiation. Following transformation into strain CL104, all of the resulting transformants were found to grow at 42 °C in the presence or absence of arabinose (Fig. S2, *A*–*C*) as previously seen with the *R. solanacearum* FabG1 (Fig. S1). In addition, all transformants grew better in the absence than in the presence of arabinose suggesting that FabG overexpression is somewhat toxic (Fig. S2, *A*–*C*). These data argued strongly that these mutant *fabG* genes encode functional 3-ketoacyl-ACP reductases and that the S138-Y151-K155 motif is not the *E. coli* FabG catalytic center. To strengthen this argument, we constructed six additional FabG mutant plasmids: Y151S, Y151I, Y151R, K155R, K155I, and K155E. These mutant proteins all remained active *in vivo* (Fig. S2, *D* and *E*).

To exclude the possibility that combinations of two or three of these residues were essential for FabG activity, additional mutant plasmids were constructed including three double mutants (S138A/Y151F, S138A/K155A, Y151F/K155A) and the triple mutant (S138A/Y151F/K155A). Upon transformation of the resulting plasmids, pTWH38 (S138A/Y151F double mutant), pZH197 (S138A/K155A double mutant), pZH198(Y151F/K155A double mutant), and pZH199 (S138A/Y151F/K155A triple mutant), into the *E. coli* strain CL104, all conferred the ability of the mutant strain to grow in the presence or absence of arabinose at 42 °C (Fig. S2*F*).

To investigate the possibility that complementation of *E. coli* strain CL104 resulted from elevated expression of the FabG mutant proteins, three single mutant *fabG* genes, three doubly mutant genes, and one triple mutant gene were inserted into the low-copy Lac promoter expression vector pHSG575 (1–5 copies/cell) to produce plasmids: pZH201 to 209. These plasmids were introduced into *E. coli* strain CL104, and the resulting CL104 derivatives were found to grow at 42 °C as well as the CL104 derivative encoding the wildtype *fabG* plasmid (Fig. S3).

**Figure 2:**
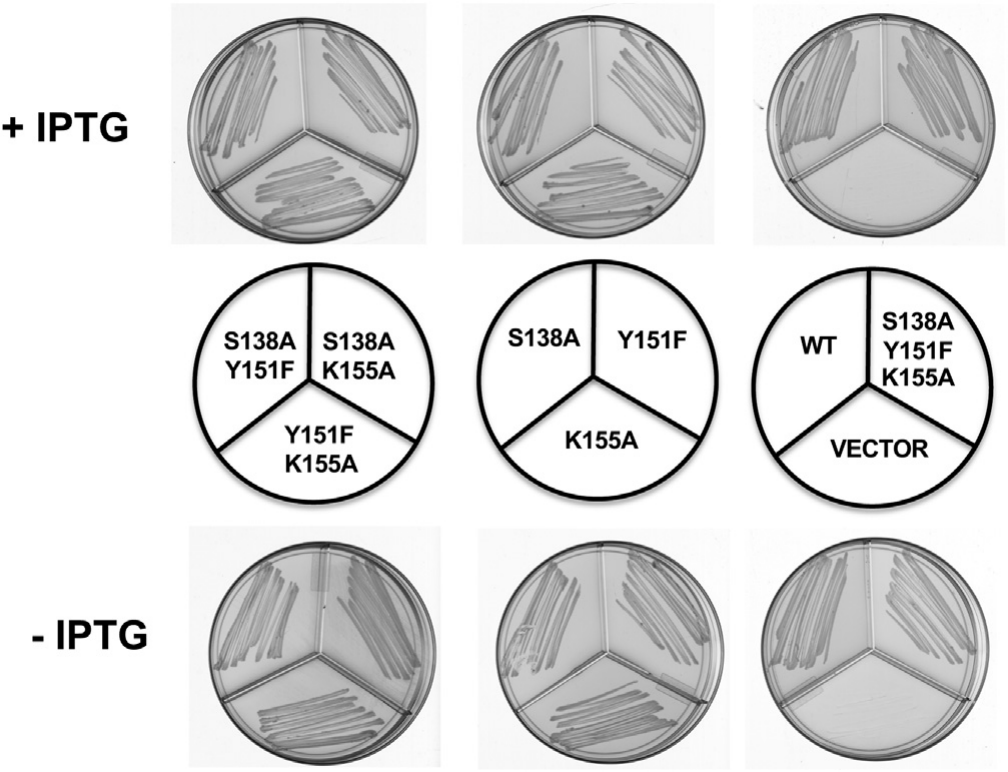
**Complementation of *S. enterica* serovar *fabG*(Ts) mutant strain CL65 by wildtype and mutant *E. coli* FabG proteins.** Growth of *Salmonella* mutant CL65 carrying the low-copy pHSG575-derived plasmids pZH201 (wildtype FabG), pZH202 (FabG S138A), pZH203 (FabG Y151F), pZH204 (FabG K155A), pZH205(FabG S138A Y151F), pZH206(FabG S138A K155A), pZH207 (FabG Y151F K155A), pZH208 (FabG S138A Y151F K155A) or the pHSG575 empty vector. + IPTG indicated addition of IPTG; − IPTG indicated no addition of IPTG.

We next tested the possibility that growth was due to homologous recombination between the plasmid-borne mutant genes and the chromosomal gene. In this scenario the two mutant alleles, the temperature-sensitive or wildtype alleles encoded on the genome and the residue substitution allele encoded by the plasmids, would recombine to give a wildtype gene on the expression plasmid. This seemed implausible based on the *R. solanacearum* results since *fabG1* and *E. coli fabG* have very different sequences. However, it remained possible when both genes are *E. coli* alleles. To eliminate this possibility we used the CL65 *fabG*(Ts) mutant of *S. enterica* serovar Typhimurium LT2 [7]. *S. enterica* LT2 is a close relative of *E. coli* and *S. enterica* proteins are generally almost identical to those of *E. coli*, whereas the coding sequences show less identity. In the case of FabG the *E. coli* and *S. enterica* LT2 proteins are 95% identical, whereas the *fabG* genes are only 88% identical. This is mainly due to third position differences (“wobble”) in the synonymous codons used by the two bacteria. Homologous recombination requires strict homology between the two strands, and recombination is severely decreased when the two strands are mismatched. This is due to two mechanisms: inefficient assembly of heteroduplexes by RecA and mismatch repair-catalyzed destruction of any heteroduplexes formed by strand exchange. Together, these processes block recombination between *E. coli* and *Salmonella typhimurium* LT2 genes (18, 19). This lack of recombination is equivalent to that given by loss of RecA function in homologous crosses but avoids the poor growth and low viability of *recA* strains. We found the plasmids encoding *E. coli* mutant proteins restored growth to the *S. enterica fabG*(Ts) strain indicating that the complementation results were not due to homologous recombination (Fig. 2).

**Figure 3:**
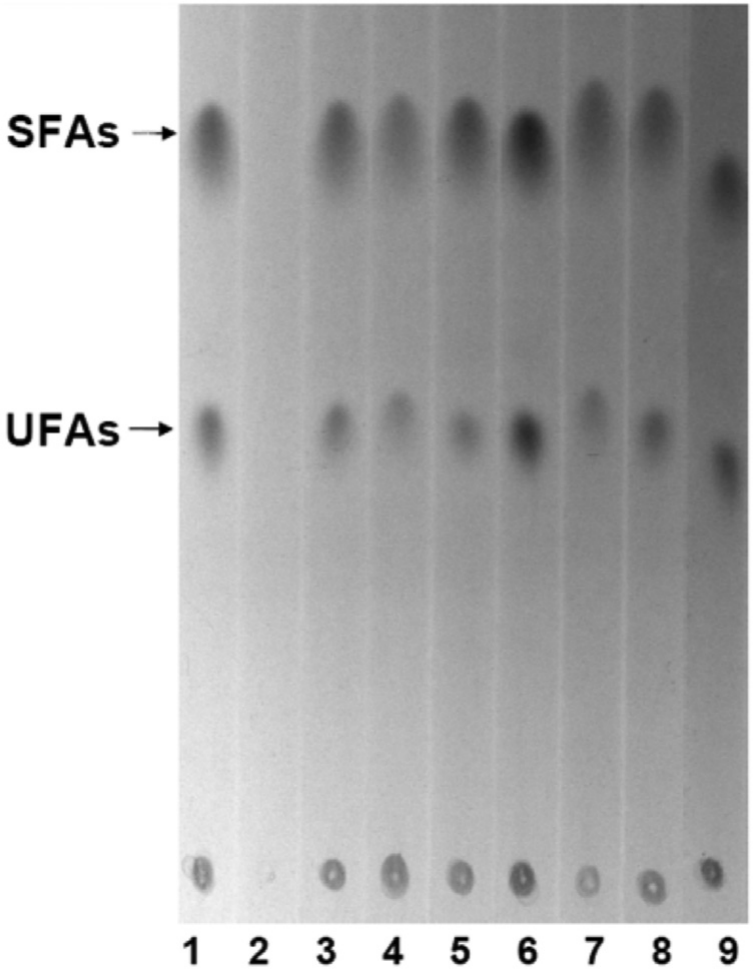
**Fatty acid biosynthesis from [1-**^**14**^**C]acetate in the *E. coli fabG*(Ts) mutant strain CL104 carrying *fabG* mutant genes at the nonpermissive temperature.** Phosphorimage of argentation thin-layer chromatographic analysis of [1-^14^C]acetate-labeled *E. coli fabG*(Ts) strain CL104 carrying the pZH201 (wildtype FabG gene, lane 1), pZH200 (pHSG575, lane 2), pZH208 (FabG S138A, Y151F, and K155A triple mutant, lane 3), pZH206 (FabG S138A and K155A double mutant, lane 4), pZH205(FabG S138A and Y151F double mutant, lane 5), pZH207(FabG Y151F and K155A double mutant, lane 6), pZH202 (FabG S138A, lane 7), pZH203 (FabG Y151F, lane 8), pZH204 (FabG K155A, lane 9) plasmid. The migration positions of the methyl esters of the fatty acids are shown. SFA, saturated fatty acid ester; UFA, unsaturated fatty acid ester.

We next compared the fatty acid synthesis profiles of strain CL104 containing wildtype FabG with those of the CL104 derivatives encoding the FabG mutant proteins grown at 42 °C. Cultures were labeled by [^14^C]acetate incorporation and the fatty acid methyl esters separated by argentation thin-layer chromatography. Our data showed that, excepting strain CL104 carrying the empty vector pHSG575, all CL104 derivatives containing either the wildtype FabG or mutant FabG proteins synthesized fatty acids normally (Fig. 3). Taken together, our data indicated that the S-Y-K triad is not the catalytic center of *E. coli* FabG.

### Characterization of the FabG mutant proteins *in vitro*

A concern in characterization of the FabG mutant proteins was that the purified tetramers could contain low levels of chromosomally encoded wildtype or temperature-sensitive mutant subunits, which might somehow bestow activity on the plasmid-encoded mutant subunits. We eliminated this possibility using two complementary approaches. We first expressed the amino terminal hexahistidine-tagged wildtype and mutant FabG proteins in the phage T7 RNA polymerase pET-28b vector system in *E. coli* strain BL21(DE3). To avoid the possibility of contamination with wildtype FabG, the mutant (and wildtype) FabG proteins were purified under denaturing conditions as described in Experimental procedures and refolded on the purification column. FabG refolded with high efficiency (probably owing to spacing of the proteins on the columns preventing aggregation); the refolded wildtype protein had full activity.

In later work we used the pQE2 vector, which allowed high-level expression in the *S. enterica fabG*(Ts) strain CL65 (7), and purified the proteins by denaturation–renaturation as given above. Use of this expression system had the advantage of precluding homologous recombination, and since each protein contains a lysine residue absent in the other protein (K72 of *E. coli* FabG, K43 of *S. enterica* FabG) this provided an assay to detect the presence of any *S. enterica* protein in our purified mutant protein preparations. This was done by trypsin digestion of the proteins followed by HPLC peptide separation and tandem mass spectral analyses of the recovered peptides. Analyses using the exponentially modified Protein Abundance Index, which denotes the ratio of observed to observable peptides, showed that the Y151F preparation contains less than 0.5% of the *S. enterica* FabG, whereas the S138A and K155A preparations contain only about 0.14% and 0.19% *S. enterica* FabG, respectively. The triple mutant *E. coli* FabG contained only 0.6% of the *S. enterica* FabG. In each case at least 93% of the possible peptides were observed experimentally.

**Figure 4:**
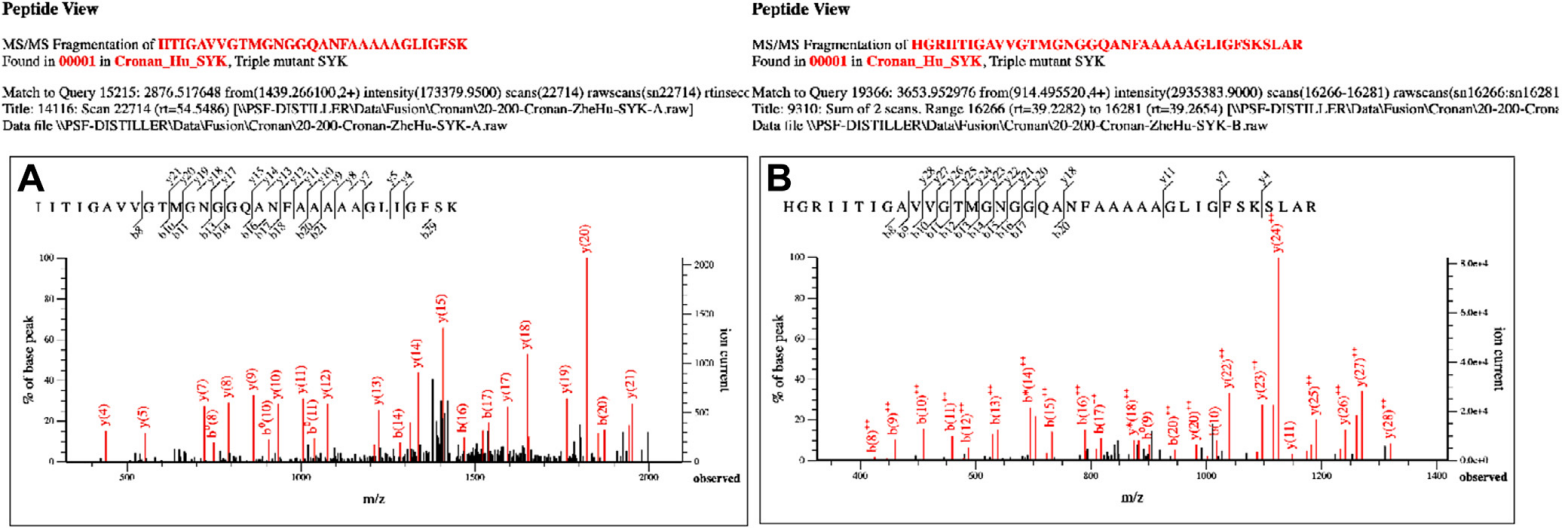
**Tandem MS sequencing of triple mutant FabG peptides.** The spectra were chosen based on Mascot quality (141 in *A*, 73 in *B*) and coverage from the amino (*b* ions) and carboxyl (*y* ions) ends of the peptide. *A*, the I133 to K163 peptide. *B*, the H130 to R167 peptide due to partial trypsin digestion. Note that MS/MS does not degrade peptides sequentially from the ends but rather somewhat randomly such that *b* and *y* ions overlap. The fragments shown in *red* are those used in identification.

**Figure 5:**
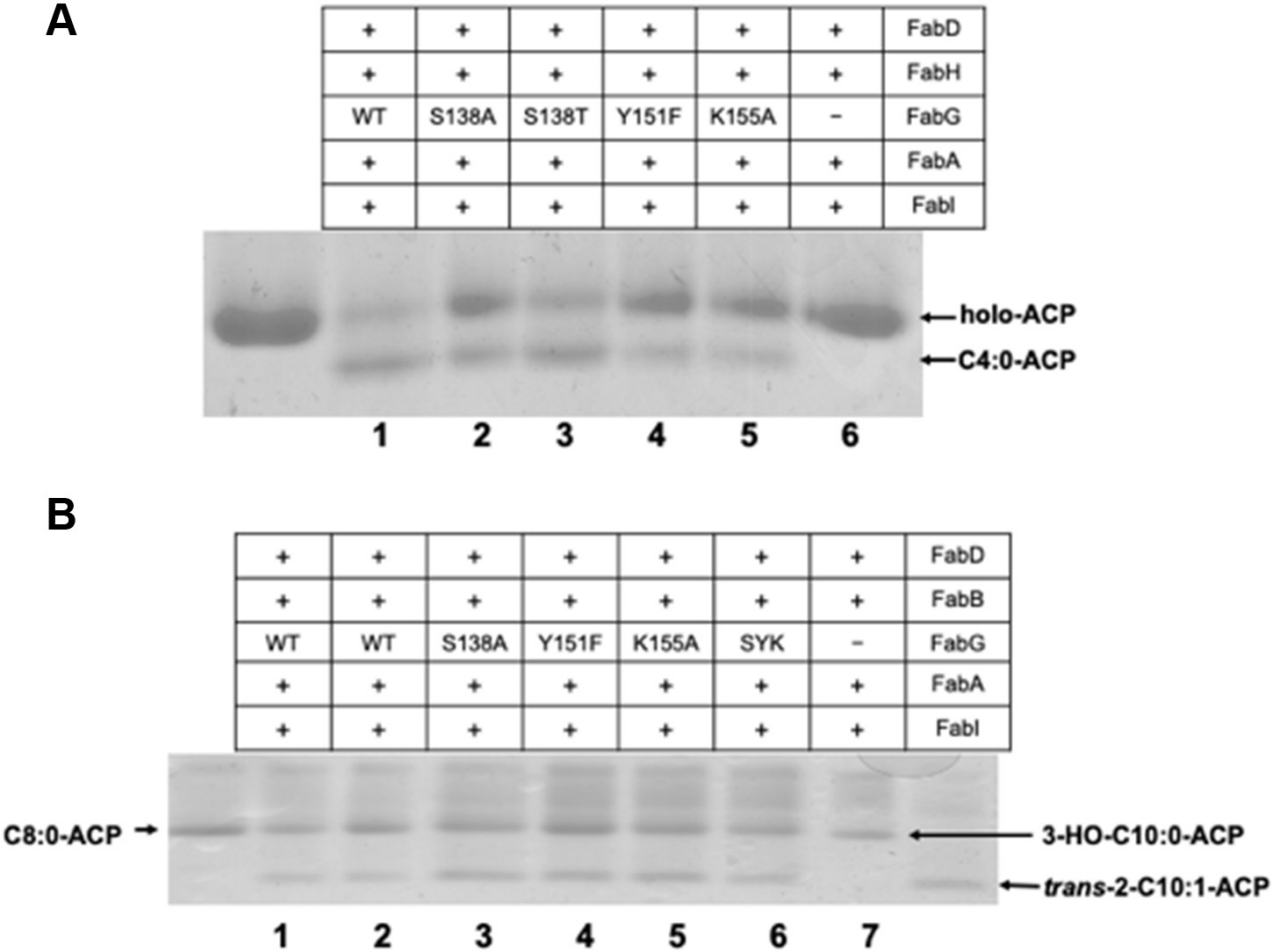
**Function of mutant FabG proteins in fatty acid synthesis.***A*, the initial cycle of fatty acid synthesis was reconstructed *in vitro* using a combination of FabH, FabG (or a FabG single mutation protein), FabA, FabI, ACP, NADH, and NADPH as cofactors and malonyl-ACP plus acetyl-CoA as substrate to produce butyryl-ACP. Lane 1, wildtype FabG; lane 2, FabG S138A; lane 3, S138T; lane 4, FabG Y151; lane 5, FabG K155A; lane 6, FabG omitted. Analyses of the three double mutants and the triple mutant are given in Experimental Information Fig. S6. *B*, elongation of octanoyl-ACP. The elongation reaction mixtures contained FabB, FabG (or a FabG single mutation protein), FabA, malonyl-ACP plus octanoyl-ACP as substrates and NADH and NADPH as cofactors. Lanes 1 and 2, FabG wildtype purified from *E. coli* or *S. enterica*, respectively; FabG K155A; lane 3, FabG S138A; lane 4, FabG Y151F; lane 5, FabG K155A; lane 6, the triple mutant S138A/Y151F/K155A FabG; and lane 7, FabG omitted. Excepting lane 1 these proteins were purified from *S. enterica* strain CL65. The wildtype protein in lane 1 was expressed in *E. coli* BL21(DE3). All proteins were purified by Ni chelate chromatography under denaturing conditions and refolded. Note that the upper bands marked holo-ACP probably also contain malonyl-ACP and acetyl-ACP (from decarboxylation of malonyl-ACP), which comigrate under these conditions. ACP, acyl carrier protein.

Finally, fragmentation by tandem mass spectroscopy (MS/MS) was used to sequence tryptic peptides that span the three putative active site residues of the triple mutant protein. Figure 4*A* shows MS/MS sequencing of a peptide that contained all three assigned active site residues. Fragments that thoroughly documented the Y151F and K115A substitutions were present in this spectrum, whereas cleavages to document the S138A substitution were sparse. However, a longer peptide (owing to lack of trypsin cleavage at R132 and K163) gave fragments that documented the S138A substitution (Fig. 4*B*). These data were confirmed by Sanger sequencing of the relevant portion of the triple mutant *fabG* gene (Fig. S4). These data demonstrated that all three expected residue substitutions had been made. Tandem MS sequencing of each of the singly mutant proteins also showed the expected residue substitutions (Fig. S5). Therefore, the putative active site residues had all been eliminated. These protein preparations were utilized in Figure 5 and for the kinetic analyses.

To determine the *in vitro* 3-ketoacyl-ACP reductase activities of the FabG proteins wildtype *fabG*, three *fabG* single mutants (S138A, Y151F and K155A), three double *fabG* mutants (S138A/Y151F, S138A/K155A, and Y151F/K155A), and one triple *fabG* mutant (S138A/Y151F/ K155A) were inserted into vector pET28. The amino terminal hexahistidine-tagged wildtype and mutant FabG proteins were then expressed in *E. coli* strain BL21 (DE3) and purified by nickel chelation chromatography under denaturation conditions and refolded. SDS-gel electrophoresis showed that all of the N-terminal His-tagged proteins were highly purified and had the expected molecular weights (Fig. S6A).

The enzymatic activity of the wildtype FabG and mutant FabG proteins was first tested in the initiation step of fatty acid synthesis. The reactions were constituted by sequentially adding initiation enzymes FabD, FabH, FabG or a mutant FabG (FabG S138A, FabG Y151F, and FabG K155A), FabA, and FabI, followed by analysis by conformationally sensitive gel electrophoresis (FabA and FabI were added to stabilize the short-chain products). In the absence of FabG, only the holo-ACP substrate was detected (Fig. 5*A*, lane7). After the addition of wildtype FabG (Fig. 5*A*, lane 2), FabG S138A (lane 3), FabG Y151F (lane 4), FabG K155A (lane 5), or FabG S138A/Y151F/K155A (lane 6) to the reaction, butyryl-ACP was produced. However, the activities of FabG S138A, FabG Y151F, and FabG K155A were lower than that of the wildtype FabG protein. These data showed that the mutant FabG proteins S138A, Y151F, K155A and triple mutant, like the wildtype FabG, could complete the initial cycle of fatty acid synthesis to produce butyryl-ACP.

A more direct assay of the enzymatic activities of the mutant FabG proteins in reduction of a long-chain 3-ketoacyl-ACP was performed. We synthesized malonyl-ACP and octanoyl-ACP as described in “Experimental procedures.” As expected, the incubation of FabB with malonyl-ACP and octanoyl-ACP resulted in the formation of 3-ketodecanoyl-ACP (Fig. 5*B*, lane 7) (the longer-chain species are more stable to electrophoresis). Upon the addition of NADPH, FabG (or one of the mutant FabG proteins), and EcFabA to the reaction mixture (lanes 1–6 of Fig. 5*B*), all incubations yielded the expected enoyl-ACP species. FabG converted 3-ketoacyl-ACP to 3-hydroxacyl-ACP, whereas EcFabA dehydrated 3-hydroxacyl-ACP to enoyl-ACP. Therefore, these data showed that all of the mutant FabG proteins were active with long-chain 3-ketoacyl-ACP substrates.

We also tested the function of the double mutant FabG proteins in fatty acid biosynthesis reactions *in vitro*. The data (Fig. S6) showed that all these mutant FabG proteins were active in *in vitro* fatty acid biosynthesis, although the activity of the triple mutant protein was much lower than that of the wildtype FabG in the initial cycle of fatty acid synthesis (Fig. S6).

### Kinetic analysis of the wildtype and single mutant FabG proteins

Acetoacetyl-CoA, an unnatural substrate, has often been used in place of acetoacetyl-ACP to assay the activity of FabG 3-ketoacyl-ACP reductase and to determine the kinetic constants of this enzyme (20, 21). However, we failed to detect any activity of the mutant FabG proteins using acetoacetyl-CoA as the substrate, although the wildtype FabG was active (data not shown). We therefore turned to an acyl-ACP–dependent spectroscopic assay to determine the kinetic constants of the wildtype and single mutant FabG proteins for NADPH binding. As shown in Table 1, the mutant FabG proteins Y151F (147.7 μM) and K155A (166.5 μM) had higher K_M_ values than the wildtype FabG (99.2 μM) for NADPH binding, whereas the mutant FabG protein S138A had a K_M_ value (101.5 μM) almost identical to that of the wildtype FabG. Our data also showed that the K*cat* values of these three mutant enzymes were similar to that of wildtype FabG, although all mutant FabG proteins exhibited a slightly lower activity than that of wildtype FabG. As a result, although these mutant FabG proteins had somewhat increased K_M_ values, they retained high 3-ketoacyl-ACP reductase activity.

**Table 1:** Kinetic constants for NADPH binding of the wildtype FabG and mutant forms of *E. coli* FabG

FabG	K_M_ (μM)	K*cat* (min^−1^)	K*cat*/K_M_ (μM^−1^ min^−1^)	Relative activity %
Wildtype	99.22	2.74	27.66	100
S138A	101.47	2.31	22.84	82
Y151F	147.72	2.45	16.57	59
K155A	166.45	2.31	13.89	50

K_M_ denotes the Michaelis constant, Kcat is the turnover number, K*cat*/K_M_ denotes the catalytic efficiency of the enzyme. Relative activity is the ratio of K*cat*/K_M_ value of each mutant protein to that of wildtype FabG as 100%. These proteins were purified from BL21(DE3) and purified by Ni chelate chromatography under denaturing conditions and refolded.

### Circular dichroism spectroscopy analyses of the wildtype FabG and mutant FabG proteins

Circular dichroism (CD) spectroscopy analysis is an excellent tool for the rapid determination of the secondary structure and folding properties of proteins. To investigate the effect of the mutations on the FabG structure, we investigated the changes of the physicochemical characteristics of FabG caused by three single mutant FabG proteins (S138A, Y151F and K155A) through CD spectroscopy analyses. The CD spectra of the wildtype FabG and mutant FabG proteins exhibited a characteristic signature of a helix with minima at 208 and 222 nm, respectively (Fig. S7*A*). The helical contents of the S138A, Y151F and K155A mutants and the wildtype FabG were 32.2%, 42.6%, 47.1%, and 33.2%, respectively. The helical content of the mutants Y151F and K155A was much higher than that of wildtype FabG, whereas the helical content of the mutant S138A was almost identical to that of the wildtype FabG. We also assayed the helical contents of the three double mutant FabG proteins (S138A/Y151F, S138A/K155A and Y151F/K155A) and the triple mutant FabG protein (S138A/Y151F/K155A). The helical contents of these mutant proteins were much higher than that of the wildtype FabG (Fig. S7*B*). These results, taken together, showed that the introduced mutations at the sites of S138, Y151, and K155 caused conformational changes in *E. coli* FabG.

The presence of NADPH has been suggested to affect the conformation of FabG (22). Our results (Fig. S7*B*) showed that the molar ellipticity of wildtype FabG and all mutant FabG proteins increased when NADPH (200 μM) was added. This observation is in agreement with previous studies (22).

### NADPH binding to wildtype FabG and mutant FabG proteins

**Figure 6:**
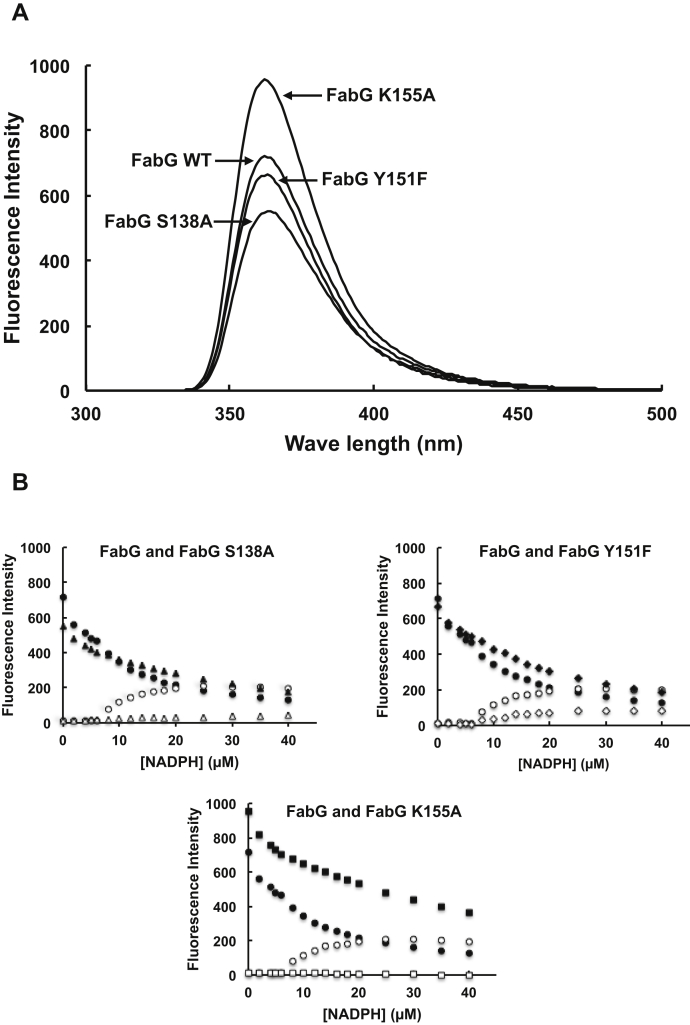
**Emission spectra for the intrinsic protein fluorescence (λ**_**max**_**363 nm) of *E*. *coli* FabG and its mutant proteins and the increase in the fluorescence of NADPH (λ**_**max**_**456 nm) upon titration of the proteins with NADPH at 20 °C.***A*, fluorescence emission spectra of FabG and its mutant proteins. The samples were excited at 280 nm. Fluorescence with a maximum at 363 nm is due to fluorescence of the single FabG tryptophan. *B*, the fractional fluorescence changes at 363 and 456 nm are plotted *versus* the varying concentrations of NADPH. The circles denote WT FabG, the *triangles* denote S138A, the *diamonds* denoteY151F, and the *squares* denote K155A. The *filled symbols* indicate quenching of tryptophan fluorescence at 363 nm; the *open symbols* indicate the enhancement of NADPH fluorescence at 456 nm. NADPH (microliter samples of 5 mM stock solutions) was added to 1 ml of FabG solution (2 μM tetramer in 3 mM Hepes, pH 7.5, 100 mM NaCl, 2 mM β-mercaptoethanol, and 10% glycerol), and the changes in fluorescence intensity were monitored between 300 and 500 nm. These proteins were purified from BL21(DE3) and purified by Ni chelate chromatography under denaturing conditions and refolded.

The spectral parameters of tryptophan fluorescence, position, shape, and intensity are dependent on the electronic and dynamic properties of the chromophore environment. Therefore, tryptophan fluorescence has been extensively applied to obtain information on the structural and dynamic properties of proteins. Tryptophan is fluorescent with an emission λ_max_ at 325 nm at pH 7.0. Buried tryptophan residues in a variety of folded proteins show a red or blue shift at the emission maximum. The *E. coli* FabG molecule contains a single tryptophan residue at position 103 and displays intrinsic fluorescence with an emission λ_max_ at 363 nm (Fig. 6*A*). To investigate the effect of the mutated S138, Y151, and K155 residues on the FabG structure, the fluorescence spectra of the wildtype FabG and mutant FabG proteins were studied by exciting the samples at 280 nm and recording the emission spectrum over a range of 300 to 500 nm. As shown in Figure 6*A*, the fluorescence spectra of the wildtype FabG and mutant FabG proteins exhibited the characteristic signature of an emission λ_max_ at 363 nm. Mutant K155A also displayed increased fluorescence intensity at 363 nm, whereas the mutant S138A and Y151F resulted in decreased fluorescence intensity.

Price *et al.* (15) observed that *E. coli* FabG exerted a conformational change when binding its cofactor NADPH and suggested that NADPH binding conferred on FabG the ability to exhibit a slight negative homotropic cooperativity (with a Hill constant of *n*_H_ = 0.9). We investigated the effect of the mutations on the NADPH binding of FabG by determining the affinity of FabG for NADPH by fluorescence spectroscopy. Owing to tryptophan fluorescence quenching at 363 nm, the fluorescence intensity of the wildtype FabG and mutant FabG proteins decreased as a function of the increasing concentrations of NADPH (Fig. 6*B*). Because of the enhancement of NADPH fluorescence at 450 nm, the fluorescence intensity of NADPH binding by wildtype FabG, S138A, or Y151F increased as a function of increasing concentrations of NADPH. The shape of the binding curve for S138A and Y151F saturated at a lower NADPH concentration, whereas the fluorescence intensity of the NADPH-binding K155A was not changed as the concentration of NADPH increased (Fig. 6*B*). Analysis of the binding curves showed that wildtype FabG and mutant proteins S138A and Y151F could bind NADPH with Kd values of 13.22, 13.89, and 15.75 μM, respectively, and that the fluorescence intensity of NADPH binding was maximized at 312.5, 56.17, and 126.58 μM, respectively. These data suggested that, although wildtype FabG and the mutant FabG S138A and Y151F had similar Kd values for NADPH binding, the mutant proteins had lower fluorescence intensity than wildtype FabG. Therefore, the mutant proteins S138A and Y151F had a decreased affinity for NADPH binding. For mutant protein K155A, no significant NADPH binding was detected at the concentrations that could be tested with this assay, suggesting that the K155A mutation may have resulted in loss of *E. coli* FabG cooperative behavior toward NADPH.

## Discussion

SDR NAD(P)(H)-dependent oxidoreductases are a very large protein family (perhaps 50,000 members). The Y151-Xaa_3_-K155 (numbering of *E. coli* FabG) segment is one of the distinct sequence motifs of SDR family enzymes (11, 13). S138 is also conserved in most SDR proteins (11). Thus, a triad of S138, Y151, and K155 was proposed to be the catalytic site of SDR enzymes (11). The first evidence supporting Y151 and K155 as SDR family catalytic site residues came from site-specific mutagenesis analyses of the *Drosophila* alcohol dehydrogenase (23, 24). When Y151 was substituted with F, H, or E and K156 was substituted with Ile, the resulting mutants lacked catalytic activity. To establish S138 as a member of a catalytically important “triad” of residues also involving Y151 and K155, Oppermann (25) exchanged S138 with A or T in the 3*β*/17*β*-hydroxysteroid dehydrogenase from *Comamonas testosteroni* and found that a Ser-to-Ala exchange at position 138 resulted in an almost complete (>99.9%) loss of enzymatic activity that was not observed with an S-to-T replacement (25). Additional evidence supporting the S138, Y151, and K155 triad as the catalytic site of SDR enzymes was provided by site-specific mutagenesis analysis of the *E. coli* 7 α-hydroxysteroid dehydrogenase (26).

However, there are examples of atypical SDRs that retain SDR structure but lack canonical active site triad residues. A recent example is a *Vibrio vulnificus* protein of unknown function that is tetrameric and has SDR topology [32]. The protein binds NADPH tightly and with extreme specificity but lacks the putative triad tyrosine and lysine residues found in FabG. The authors postulate another tyrosine-dependent site, but since the substrate of the reaction is unknown, testing of the postulated site was precluded. Another example of an atypical SDR is human peroxisomal enoyl CoA reductase where the active site Y is replaced by an F residue (27). To our knowledge, although various alternative active site residues have been proposed for a few atypical SDR proteins, we can find no tests of these hypotheses. A problem is that many proteins annotated as atypical SDRs result from high-throughput crystallography consortia. The proteins are assigned only by their conserved structures and lack of canonical active site residues. The reactions catalyzed are unknown.

We also observed that the *Pseudomonas aeruginosa fabV* gene, encoding a new isoform of SDR enoyl-ACP reductase, remained able to complement an *E. coli fabI* temperature-sensitive mutant strain upon substitution of Y235 with F or K244 with M (28).

We considered mutating the three FabG Tyr residues other than Y155, but these residues are located well away from the NADPH cofactor and two of the three seem involved in interface interactions (FabG is a dimer of dimers, hence two different interfaces are present). The Ts mutants we isolated previously all mapped in interface regions showing the very strong interface dependence of this enzyme activity (17). The third Tyr is on the surface and exposed to solvent. Attempts to deduce a mechanism are hindered by the lack of information on the location of the acyl chain of the 3-ketoacyl-ACP in the structure. Since FabG is required for elongation of C4 to C16 acyl chains, we would expect to see a long hydrophobic tube or cleft in the structure (as seen for other fatty acid elongation cycle enzymes) but the prior crystal structures give no hints of how acyl chains are bound. A known acyl chain binding site would focus the active site hunt. Untargeted mutagenesis of a plasmid-borne *fabG* seems likely to be unrewarding because the interfaces are significantly larger targets for mutation than the active site.

*E. coli* FabG seemed a typical example of an SDR protein and contains the highly conserved triad of S138-Y151-K155 (8) and the triad was assumed to be the catalytic site of FabG. Previously, Price and colleagues (8, 15) determined the structure of FabG and the mutant FabG Y151F and suggested that cofactor binding induced a conformational rearrangement of FabG, causing the triad of S138-Y151-K155 to establish a catalytically competent active site. In addition, the mutant FabG Y151F lost the ability to reduce acetoacetyl-CoA *in vitro* (8, 15). In this regard, recall that none of our mutant proteins could reduce acetoacetyl-CoA, although they were active with the natural substrate, acetoacetyl-ACP. Therefore, based on the acetoacetyl-CoA result Price and coworkers reported that the S138-Y151-K155 triad comprised the FabG catalytic center (8, 15). However, no additional experimental evidence was provided to support this hypothesis.

In conclusion we have demonstrated that substitution of *E. coli* FabG residues S138, Y151, and K155 with a variety of other residues failed to inactivate the enzyme. We have thoroughly characterized the S138A, Y151F, and K155A substitutions as single mutant, double mutant, and the triple mutant. All mutant sequences were confirmed by DNA sequencing of plasmids extracted from the cell pellets of cultures grown for protein production. Moreover, the relevant tryptic peptides of the S138A, Y151F, and K155A single mutant proteins plus the triple mutant peptide were sequenced by tandem mass spectroscopy (MS/MS). The MS/MS results directly demonstrated that each of the designed residue substitutions had been made. We have ruled out contamination of our purified proteins with recombinant species resulting from genetic recombination or formation of tetramer species containing a mixture of plasmid and chromosomally encoded proteins. The *fabG*(Ts) strain CL65 of *S. enterica* serovar LT2 prevented genetic recombination and also provided a heterologous host with a distinguishable FabG sequence that could be used to assay for mixed tetramers by tandem mass spectroscopy. Urea denaturation and subsequent renaturation of the column-bound proteins also precluded assay of mixed tetramers. We believe that we have eliminated each of the possible pitfalls and therefore conclude that *E. coli* FabG does not catalyze the reduction of 3-ketoacyl-ACPs using the classical S-Y-K triad.

## Experimental procedures

### Bacterial strains and growth media

The *E. coli* strains and plasmids are listed in Table S1. Luria-Bertani (LB) medium was used as the rich medium for the *E. coli* strains. The phenotypes of the *E. coli fab* strains were assessed on rich broth medium (29). Antibiotics were used at the following concentrations (in μg/ml): sodium ampicillin, 100; kanamycin sulfate, 30; chloramphenicol, 30; and gentamicin, 20. L-Arabinose was used at a final concentration of 0.02%. Isopropyl-β-D-thiogalactoside (IPTG) was used at a final concentration of 1 mM, and 5-bromo-4-chloro-3-indolyl-β-D-galactoside (X-Gal) was used at a final concentration of 20 μg/ml.

### Recombinant DNA techniques and construction of plasmids

The *E. coli fabG* gene was amplified using the genomic DNA of *E. coli* MG1655 as the template. The PCR amplification was performed with *Pfu* DNA polymerase and two primers, EcfabG-F-NdeI and EcfabG-R-HindIII, as listed in Table S2. The PCR product was inserted into the T-vector plasmid pMD19 to produce plasmid pTWH1. All mutated *fabG* genes were prepared by overlapping PCR using pTWH1 as the template, and the mutagenic primers are listed in Table S2. The mutated *fabG* genes were inserted into the T-vector plasmid pMD19. All cloned *fabG* sequences were confirmed by sequencing performed by Shanghai Sangon, Inc. The *fabG* gene fragments digested from the T-vectors with NdeI and HindIII were gel purified and ligated into pBAD24M (28), pET28(b) or pQE2, which was digested with the identical enzymes, to produce the plasmids listed in Table S1. To construct the pHSG575 series of *fabG* expression vectors, the *fabG* genes were digested from the pBAD24M series *fabG* expression vectors by BamHI and HindIII and were inserted into the identical sites of pHSG575. To facilitate screening, a 750-bp gentamicin resistance cassette was obtained from plasmid p34s-Gm by HindIII digestion and inserted into the identical sites of the above intermediate plasmids.

### Expression and purification of hexahistidine-tagged proteins

The pQE2 plasmids encoding *fabG* mutant proteins were transformed into *S. enterica* serovar LT2 mutant strain CL65 for expression of the mutant FabG proteins at a high level upon IPTG induction (protein production by the phage T5 pQE promoter rivals that of the pET system). The FabG wildtype and mutant proteins were purified as follows. In total, 1 l of LB cultures of FabG expression strains was collected by centrifugation (4000*g*, 4 °C, 20 min) after IPTG induction at 30 °C for 3 h. The cells were suspended in 10 ml of lysis buffer (100 mM NaH_2_PO_4_·2H_2_O, 300 mM NaCl, pH 8.0, and 8 M urea) and were disrupted by two passages through a French press cell at maximum pressure. The soluble cell extract was obtained by centrifugation at 20,000*g* for 20 min. The supernatant was applied to a Ni-NTA agarose-containing column and mixed gently for 1 h at 4 °C. The bottom cap of the column was removed, and the flow-through was collected. After washing with buffer (100 mM NaH_2_PO_4_, 300 mM NaCl, pH 8.0) containing a step-gradient concentration of urea (8–0 M) at 4 °C, the lysate-resin was washed twice using an identical buffer plus 20 mM imidazole. The His-tagged proteins were eluted with the elution buffer (100 mM NaH_2_PO_4_, 300 mM NaCl, and 250 mM imidazole, pH 8.0) and then dialyzed overnight at 4 °C against 4 L of buffer (100 mM NaH_2_PO_4_, 300 mM NaCl, pH 8.0). The purified proteins were stored at −80 °C with addition of 20% glycerol at final concentration. The mutant proteins are also expressed and purified from BL21(DE3) using as described above. We also purified the *E. coli* fatty acid synthetic proteins (FabD, FabH, FabG, FabZ, FabB, FabI, and holo-ACP) and *Vibrio harveyi* acyl-ACP synthetase (AasS) as described (28, 30).

### Analysis of fatty acid compositions

The ability of the FabG mutants to restore fatty acid synthesis was tested in the *E. coli fabG*(Ts) mutant strain CL104. Briefly, the IPTG-inducible vector pHSG575-carrying genes encoding FabG or FabG mutant proteins was transformed into CL104. The cultures were grown at a permissive temperature, induced with IPTG, and shifted to 42 °C. After the incubation, [1-^14^C]acetate was added as described (28). The labeled fatty acids were extracted, analyzed by argentation thin-layer chromatography (TLC), and quantified by phosphorimaging (31).

### ACP-dependent gel reconstitution assay of FabG activity

Malonyl-ACP was synthesized from holo-ACP and malonyl-CoA using *E. coli* FabD. Octanoyl-ACP was synthesized from octanoic acid, ATP, and *E. coli* holo-ACP by use of *V. harveyi* acyl-ACP synthetase as described (32). The ability of FabG or FabG mutant proteins to function in fatty acid synthesis was assessed in reactions reconstituted by the sequential addition of purified *E. coli* fatty acid synthetic enzymes and cofactors in sodium phosphate (pH 7.0) as described (28, 30). The reaction products were separated by conformationally sensitive gel electrophoresis (33).

### Spectrophotometric assay of FabG activity

The spectrophotometric assay for FabG activity was measured by the oxidation rate of NADPH at 340 nm. Two substrates (acetoacetyl-CoA and acetoacetyl-ACP) were used for the assay. When using acetoacetyl-CoA as a substrate, the reaction mixture contained 0.5 mM acetoacetyl-CoA, 0.2 mM NADPH, 10 μg of FabG (or a mutant FabG), and 0.1 M sodium phosphate buffer with a pH of 7.4 in a final volume of 300 μl. When using acetoacetyl-ACP as a substrate, the mixture contained 100 μM ACP, 1 mM β-mercaptoethanol, 500 μM malonyl-CoA, 500 μM acetyl-CoA, varying concentrations of NADPH, 0.5 μg of purified *E. coli* FabD, 0.5 μg of purified EcFabH in 0.1 m sodium phosphate buffer (pH 7.0), and 5 μg of FabG (or a mutant FabG) protein in a final volume of 100 μl. The kinetic constants were determined using GraphPad Prism software, version 4.

### Tandem mass spectroscopy of FabG peptides

Protein samples were digested with trypsin (Promega sequencing grade modified) prior to LC-MS/MS analysis by the Protein Sciences Facility of the University of Illinois Carver Biotechnology Center. Trypsin cuts proteins at the carboxyl side of K or R residues. LC-MS/MS analysis was performed on a Thermo Scientific Fusion Orbitrap mass spectrometer coupled to a Dionex Ultimate 3000 RSLC nano UHPLC. The separation was performed on a Thermo Scientific Acclaim PepMap RSLC (75 μm × 15 cm) C18, 2-μm, 100-Å column at 300 nl/min. The gradient (A: 0.1% formic acid in water, B: 0.1% formic acid in acetonitrile) was run over 76 min with the following compositions: 0 to 6 min 1% to 4% B, 6 to 51 min 4% to 35% B, 51 to 56 min 35% to 50% B, 56 to 60 min 50% to 90% B, 60 to 64 min 90% B, 64 to 66 min 90% to 2% B, 66 to 76 min 2% B. In each case the volume was made up to 100% with A, the Fusion Tribrid settings were in the Peptide mode and the Universal method with resolution of 120,000, precursor mass range 300 to 1600, RF lens 60%, AGC 2.0e5, Injection Time 100 ms, Intensity threshold 5.0 e3, Charge state 1 to 7, Exclude after 2 times in 30 s for 60 s, Mass tolerance ±10 ppm, Data Dependent MS^2^, collision induced dissociation, Isolation 1.6 *m/z*, CID activation, Collision energy 35%, IT detection in Rapid Scan mode, AGC 1.0e4, All parallelizable time, maximum Injection time 60 s, 3 s/cycle.

Raw data were processed into peak lists using Mascot Distiller 2.7.1.0 (Matrix Science) and searched using Mascot 2.7.0.1 against a custom database composed of two entries (mutant sequence and wildtype host sequence). Mascot search settings included three missed cleavages, no fixed modifications, oxidation (Met) variable modifications, precursor ion mass tolerance ±10 ppm, fragment ion mass tolerance ±0.3 Da, and the threshold for accepting individual spectra of 13. The mass spectrometry proteomics data have been deposited to the ProteomeXchange Consortium *via* the PRIDE (34) partner repository with the dataset identifier PXD023486 and 10.6019/PXD023486. The wildtype *E. coli* K-12 and *S. enterica* LT2 FabG sequences were taken from UniProtKB/Swiss-Prot entries P0AEK2 and P0A2C9, respectively.

### Circular dichroism measurements

The CD spectra of FabG and mutant FabG proteins were obtained with a Chirascan (Applied Photophysics Limited) at 25 °C using a 1.0-nm bandwidth, 1-mm cell, 1.0-nm step, 0.5-s time-per-point, and 1.0-min time interval. The CD spectra were measured at an enzyme concentration of 4 μM in 50 mmol/L phosphate-buffered saline (PBS, pH 7.5), and when required, a final concentration of 200 μM NADPH was added to each sample. The results were expressed as molar ellipticity (θ) deg cm^2^ dmol^−1^. The values were normalized by subtracting the baseline recorded for the buffer under similar conditions.

### Fluorescence titration of FabG-NADPH binding

Equilibrium binding of a ligand to FabG or its mutant proteins was measured by fluorescence titration at 20 °C using a Jobin-Yvon Horiba spectrofluorometer and band-passes of 3 and 5 nm as the excitation and emission monochromators, respectively. The fluorescence spectrum of FabG was studied by exciting the samples at 280 nm and recording the emission spectrum over the range of 300 to 500 nm. For the NADPH association constant calculation, the samples were excited at 363 nm. Aliquots of 3 μl of NADPH (from stock solutions of 100 μM) were added to 4 μM FabG in 50 mM potassium phosphate buffer at a pH of 7.5. The solution was mixed after the addition of each aliquot, and the fluorescence intensity in the 400- to 500-nm region was recorded as the average of three readings.

## Data availability

All data are in this article or Supporting Information excepting the tandem mass spectroscopy data, which have been deposited into the PRIDE database (Project Name: 20-175-Cronan-ZheHu and 20-200-Cronan-ZHu; Project accession: PXD02348 and Project DOI: 10.6019/PXD023486). The URL for PRIDE is https://www.ebi.ac.uk/pride/.

## Supporting information

This article contains supporting information.

## Conflict of interest

The authors declare that they have no conflicts of interest with the contents of this article.
