# Supplementary material for: Escherichia coli FabG 3-ketoacyl-ACP reductase proteins lacking the assigned catalytic triad residues are active enzymes
Source: J Biol Chem. 2021 Feb 3;296:100365. doi: 10.1016/j.jbc.2021.100365 (PMC7973133; doi:10.1016/j.jbc.2021.100365)
Supplement: Supplemental Figures S1–S7 and Tables S1–S2 [file mmc1.pdf]

Supporting Information

Escherichia coli FabG 3-ketoacyl-ACP reductase proteins lacking the assigned catalytic triad residues are active enzymes.

Zhe Hu, Jincheng Ma, Wenhua Tong Lei Zhu, Haihong Wang and John E Cronan

Table S1. Strains and plasmids used in this work

**Strains or plasmids**	**Relevant characteristic(s)**	**Source or reference**
***E. coli* strains**		
**DH5α**	F-, *deoR*, *endA1*, *gyrA96*, *hsdR17* (rk-mk+), *recA1*, *relA1*, *supE44*, *thi-1*, del(l*acZYA*-*argF*)U169, (Phi80l*acZ*del*M15*)	Lab collection
**BL21 (DE3)**	*F-, lon-11, Δ(ompT-nfrA)885, Δ(galM-ybhJ)884, λDE3 [lacI, lacUV5-T7 gene 1, ind1, sam7, nin5], Δ46, [mal+]K-12(λS), hsdS10*	Lab collection
**CL104**	*fabG*(Ts) *panD*, Cm^r, Tet^r, Km^r	[1]
**MG1655**	Wild type	Lab collection
S. enterica* serovar *Typhimurium		
**CL65**	*fabG*(Ts) *fabF::*Kan of LT2	[1]
**Plasmids**		
**pMD19**	Amp^r, T-vector	Takara

**pBAD24M**	Amp^r; NcoI site of expression vector pBAD24 changed to a NdeI site	[2]
**pET-28b**	Km^r, expression vector	Novagen
**p34s-Gm**	Amp^r; Gm resistance cassette-carrying vector	[3]
**pHSG575**		[4]
**pQE2**	Amp^r, Expression vector	Lab collection
**pTWH1**	Amp^r, *fabG* in pBAD24M	This study
**pTWH3**	Km^r, *fabG* in pET-28b	This study
**pTWH4**	Km^r, *fabG* S138A in pET-28b	This study
**pTWH5**	Km^r, *fabG* Y151F in pET-28b	This study
**pTWH6**	Km^r, *fabG* K155A in pET-28b	This study
**pTWH22**	Amp^r, *fabG* S138A mutant in pBAD24M	This study
**pTWH23**	Amp^r, *fabG* S138T mutant in pBAD24M	This study
**pTWH24**	Amp^r, *fabG* Y151F mutant in pBAD24M	This study
**pTWH25**	Amp^r, *fabG* Y151H mutant in pBAD24M	This study
**pTWH29**	Amp^r, *fabG* K155T mutant in pBAD24M	This study
**pTWH30**	Amp^r, *fabG* K155A mutant in pBAD24M	This study
**pTWH31**	Amp^r, *fabG* Y151R mutant in pBAD24M	This study
**pTWH32**	Amp^r, *fabG* Y151I mutant in pBAD24M	This study
**pTWH33**	Amp^r, *fabG* Y151S mutant in pBAD24M	This study

**pTWH34**	Amp^r, *fabG* K155R mutant in pBAD24M	This study
**pTWH35**	Amp^r, *fabG* K155I mutant in pBAD24M	This study
**pTWH36**	Amp^r, *fabG* K155E mutant in pBAD24M	This study
**pTWH38**	Amp^r, *fabG* S138A/Y151F mutant in pBAD24M	This study
**pZH197**	Amp^r, *fabG* S138A/K155A mutant in pBAD24M	This study
**pZH31**	Kan^r, pET28(b)-EcFabA	Lab collection
**pZH32**	Kan^r, pET28(b)-EcFabB	Lab collection
**pZH33**	Kan^r, pET28(b)-EcFabD	Lab collection
**pZH34**	Kan^r, pET28(b)-EcFabH	Lab collection
**pZH35**	Kan^r, pET28(b)-EcFabI	Lab collection
**pZH36**	Kan^r, pET28(b)-EcFabA	Lab collection
**pZH59**	Kan^r, pET16(a)-VhAasS	Lab collection
**pZH72**	Kan^r, pET28(b)-EcAcpP	Lab collection
**pZH73**	Cm^r, pBAD34-EcAcpS	Lab collection
**pZH198**	Amp^r, *fabG* Y151F/K155A mutant in pBAD24M	This study
**pZH199**	Amp^r, *fabG* S138A/Y151F/K155A mutant in pBAD24M	This study
**pZH200**	Cm^r, Gm ^{r}, pHSG575 -Gm	This study
**pZH201**	Cm^r, Gm^r, *fabG* in pHSG575	This study
**pZH202**	Cm^r, Gm^r, *fabG* S138A in pHSG575	This study
**pZH203**	Cm^r, Gm^r, *fabG* Y151F in pHSG575	This study

**pZH204**	Cm^r, Gm^r, *fabG* K155A in pHSG575	This study
**pZH205**	Cm^r, Gm^r, *fabG* S138A/ Y151F in pHSG575	This study
**pZH206**	Cm^r, Gm^r, *fabG* S138A/ K155A in pHSG575	This study
**pZH207**	Cm^r, Gm^r, *fabG* Y151F/ K155A in pHSG575	This study
**pZH208**	Cm^r, Gm^r, *fabG* S138A/Y151F/ K155A in pHSG575	This study
**pZH209**	Amp^r, pQE2- EcFabG	This study
**pZH210**	Amp^r, pQE2- EcFabG S138A	This study
**pZH211**	Amp^r, pQE2- EcFabG Y151F	This study
**pZH212**	Amp^r, pQE2- EcFabG K155A	This study
**pZH213**	Amp^r, pQE2- EcFabG S138A Y151F K155A	This study
**pHZ214**	Km^r, *fabG* S138A Y151F in pET-28b	This study
**pHZ215**	Km^r, *fabG* Y151F K155A in pET-28b	This study
**pHZ216**	Km^r, *fabG* S138A K155A in pET-28b	This study
**pHZ217**	Km^r, *fabG* S138A Y151F K155A in pET-28b	This study

Table S2. Sequences of the PCR primers used in this work

Primer name	Primer sequence (5' to 3')
EcfabG NdeI Forward	ACATATGAATTTTGAAGGAAAAATCGCAC
EcfabG HindIII Reverse	GCAAGCTTCAGACCATGTACATCCCG
S138A Forward	CACTATCGGTGCTGTGGTTGGTACCATG
S138A Reverse	CATGGTACCAACCACAGCACCGATAGTG
Y151F Forward	GTCAGGCCAACTTCGCTGCGGCGAAAG
Y151F Reverse	CTTTCGCCGCAGCGAAGTTGGCCTGAC
K155A Forward	CTACGCTGCGGCGGCAGCGGGCTTGATC
K155A Reverse	GATCAAGCCCGCTGCCGCCGCAGCGTAG
Y151F K155A Forward	CTTCGCTGCGGCGGCAGCGGGCTTGATC
Y151F K155A Reverse	GATCAAGCCCGCTGCCGCCGCAGCGAAG

Supplementary Figures

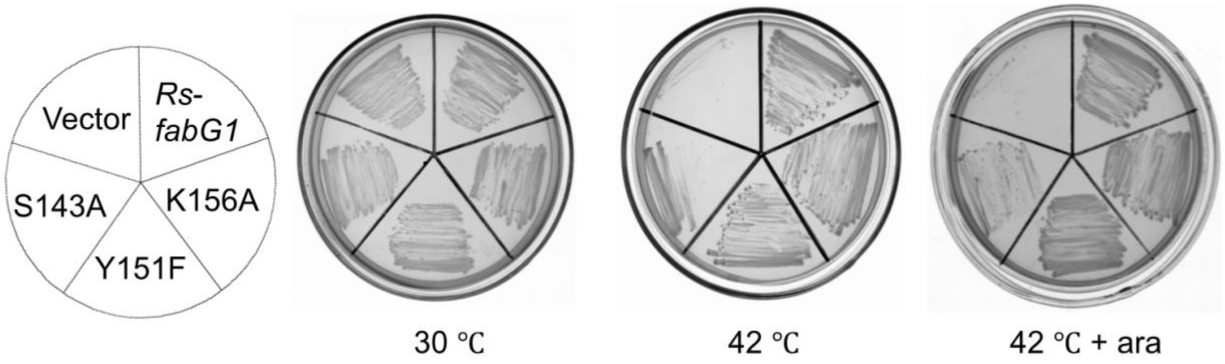


```
Rs 5 LNNQVALVTGASRGIGRAIALELARQGATVVGTATSEAGAQAISAYFAEAGVKGAGVVLN 64
Ec 3 FEGKIALVTGASRGIGRAIAETLAARGAKVIGTATSENGAQAISDYL---GANGKGLMLN 59

 65  VNDAARCEAVIDEAIKTHGGLNILVNNAGITQDNLAMRMKDDEWMAVIDTNLSAVFRLSR 124
 60  VTDPASIESVLEKIRAEFGEVDILVNNAGITRDNLLMRMKDEEWNDIIETNLSSVFRLSK 119

 125 AVLRPMMKARGGRIINITSVVGSAGNPGQANYAAAKAGVEGMARAMAKEIGSRNITVNSV 18
 120 AVMRAMMKKRHGRIITIGSVVGTMGNGGQANYAAAKAGLIGFSKSLAREVASRGITVNVV 179

 185 APGFIDTDMTKVLSDEQHTALKAQIPLGRLGMPEDIANAVAFLASPAAGYITGATLHVNG 244
 180 APGFIETDMTRALSDDQRAGILAQVPAGRLGGAQEIANAVAFLASDEAAYITGETLHVNG 239

 245 GMYM 248
 240 GMYM 243
```

Fig. S1. Mutagenesis of putative active site residues of *Ralstonia solanacearum* GMI1000 FabG1.

Upper panel: The *fabG1* gene was amplified from *R. solanacearum* GMI1000 genomic DNA and inserted into plasmid pBAD24M as described for *E. coli fabG*. Following introduction of the mutation of putative active site residues, the mutant and wild type plasmids were transformed into the *E. coli fabG(Ts)* strain CL104. Single transformant colonies were then streaked on the media given above and incubated at the temperatures given. The methods used were essentially those of Fig. S2. Lower panel: Alignment of

R. solanacearum FabG (Rs) with *E. coli* FabG (Ec). Identical residues are in red and the putative active site residues are marked with a larger blue font. Note that *R. solanacearum* is a β-proteobacterium only distantly related to *E. coli.* The two DNA sequences are only 52% conserved which preclude homologous recombination. The *R. solanacearum fabG* has a higher GC content (70%) than *E. coli fabG* (52%) which seems likely to compromise its translation efficiency in *E. coli*.

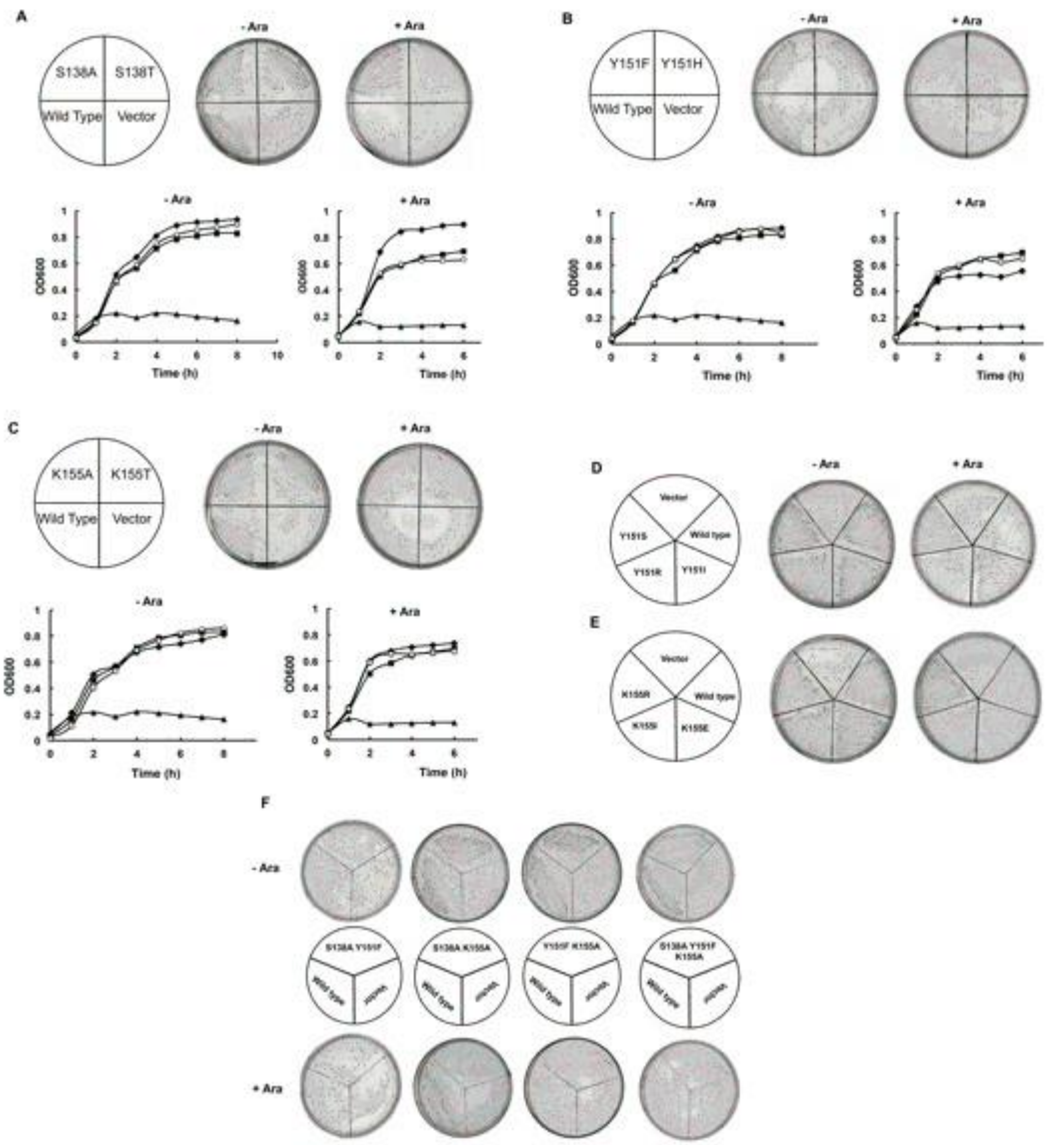

Fig. S2. Growth phenotypes of *E.coli fabG*(Ts) mutant strain CL104 carrying plasmids encoding *fabG* single mutant genes at the nonpermissive temperature.

Panel A, Growth of *E.coli* mutant CL104 carrying the pBAD24M-derived plasmids pTWH1 (wild-type FabG), pTWH22 (FabG S138A), pTWH23 (FabG S138T) or pBAD24M empty vector. (▲), CL104 carrying plasmid pBAD24M; (■), CL104 carrying plasmid pTWH1; (○), CL104 carrying pTWH22; (◆), CL104 carrying plasmid pTWH23. Panel B, Growth of *E.coli* mutant CL104 carrying the pBAD24M-derived plasmids

pTWH1, pTWH24 (FabG Y151F), pTWH25 (FabG Y151H) or pBAD24M empty vector. (▲), CL104 carrying plasmid pBAD24M; (■), CL104 carrying plasmid pTWH1; (○), CL104 carrying pTWH24; (◆), CL104 carrying plasmid pTWH25. Panel C, Growth of *E.coli* mutant CL104 carrying the pBAD24M-derived plasmids pTWH1, pTWH29 (FabG K155T), pTWH30 (FabG K151A) or pBAD24M empty vector. (▲), CL104 carrying plasmid pBAD24M; (■), CL104 carrying plasmid pTWH1; (○), CL104 carrying pTWH29; (◆), CL104 carrying plasmid pTWH30. Growth curves of strain grown in LB with arabinose. Panel D, Growth of *E.coli* mutant CL104 carrying the pBAD24M-derived plasmids pTWH1, pTWH31 (FabG Y151R), pTWH32 (FabG Y151I), pTWH33 (FabG Y151S) or pBAD24M empty vector. Panel E. Growth of *E.coli* mutant CL104 carrying the pBAD24M-derived plasmids pTWH1, pTWH34 (FabG K155R), pTWH35 (FabG K155I), pTWH36 (FabG K155E) or pBAD24M empty vector. Panel F, Growth of *E.coli* mutant CL104 carrying the pBAD24M-derived plasmids pTWH1, pTWH38 (FabG S138A Y151F), pZH197 (FabG S138A K155A), pZH198(FabG Y151F K155A), pZH199(FabG S138A Y151F K155A) or pBAD24M empty vector.+ Ara indicated addition of arabinose; - Ara indicated without addition of arabinose.

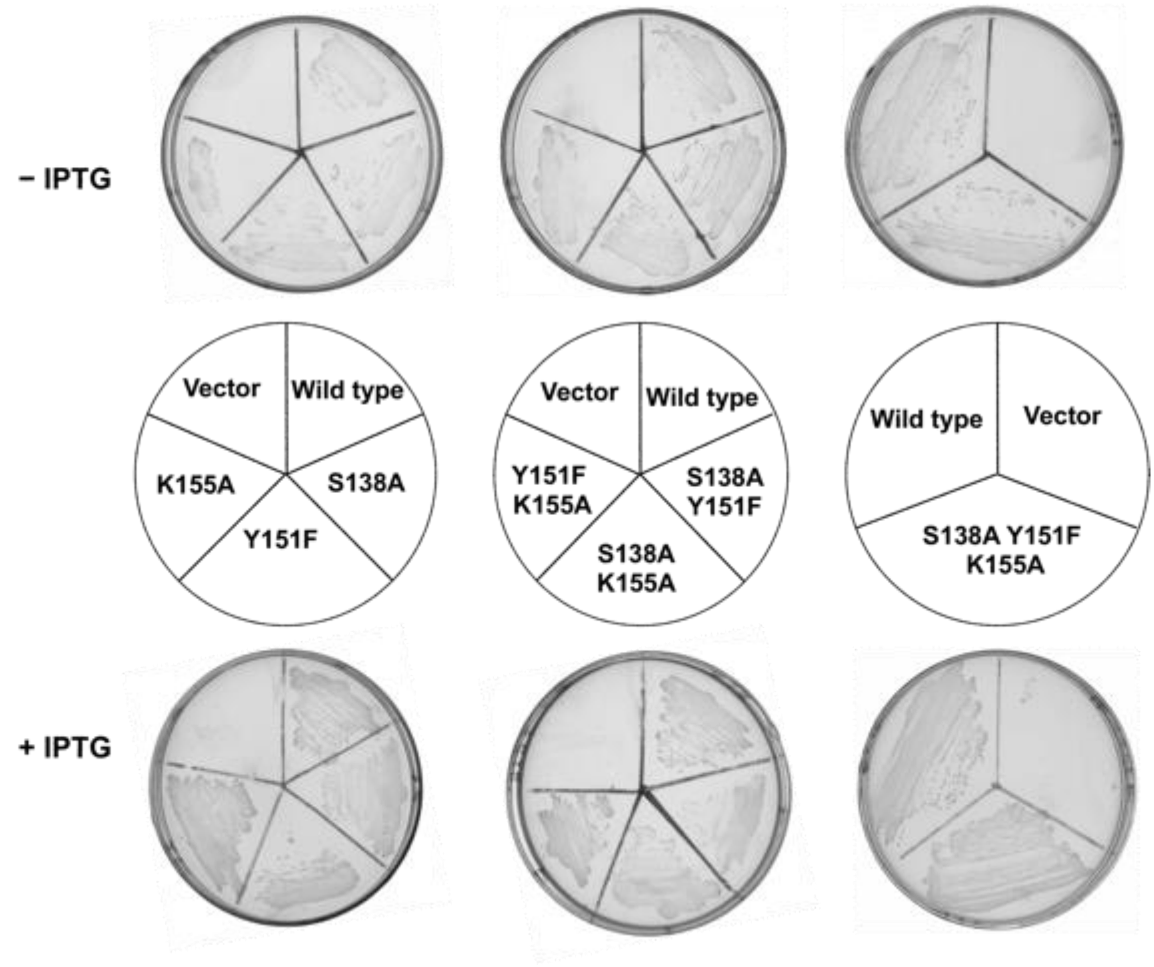

Fig. S3. Growth phenotypes of *E.coli fabG*(ts) mutant strain CL104 carrying low copy plasmids encoding *fabG* mutant genes at nonpermissive temperature. Growth of *E.coli* mutant CL104 carrying the pHSG575-derived plasmids pZH201(wild-type FabG), pZH202(FabG S138A), pZH203(FabG Y151F), pZH204(FabG K155A), pZH205(FabG S138A Y151F), pZH206(FabG S138A K155A), pZH207(FabG Y151F K155A), pZH208(FabG S138A Y151F K155A),or pHSG575 empty vector. + IPTG indicated addition of IPTG; - IPTG indicated without addition of IPTG.

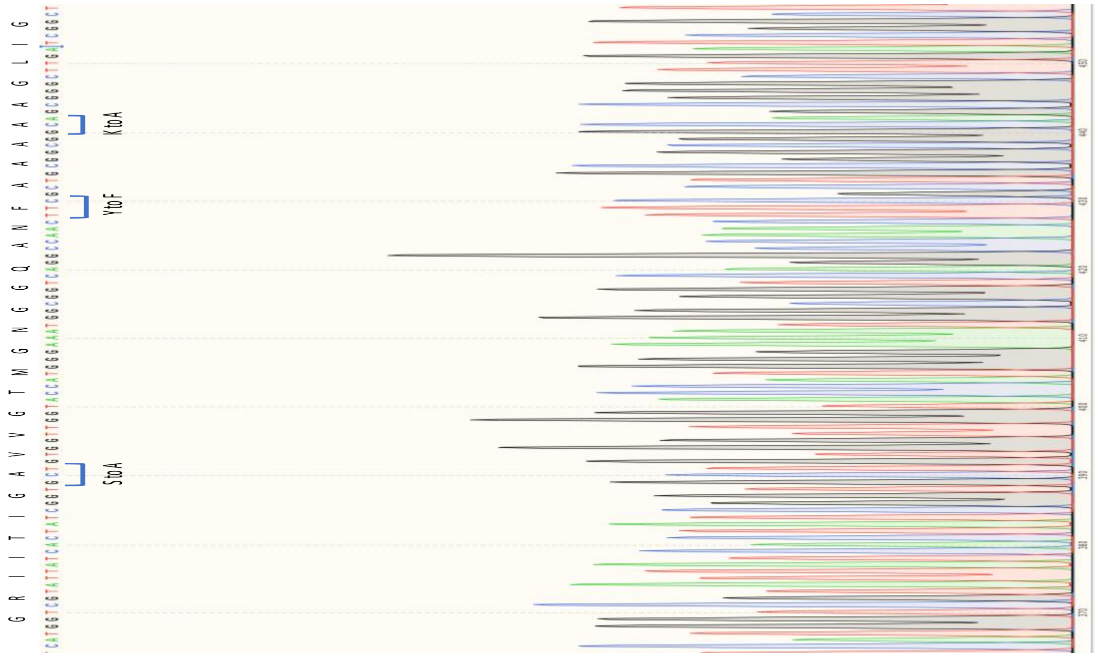

Fig. S4. Annotated Applied Biosystems Sanger sequence file of the relevant section of the triple mutant f*abG* gene. The sequenced plasmid was extracted from the cells used to produce the protein in strain CL65.

(A) S138A

P Peptide View

N MS/MS Fragmentation of **IITIGAVVGTMGNGGQANYAAAK**
F Found in **00003** in **Cronan_Zhe_FabG**, Hu Cronan EcFabG-S mutant

N Match to Query 10384: 2192.125248 from(1097.069900,2+) intensity(39716.2710) scans(9128) rawscans(sn9128) rtinseconds(1781.0884) index(3534)
T Title: 3535: Scan 9128 (rt=29.6848) [E:\Data\Fusion\Cronan\20-175-Cronan-Zhe-S-R.raw]
D Data file E:\Data\Fusion\Cronan\20-175-Cronan-Zhe-S-R.raw

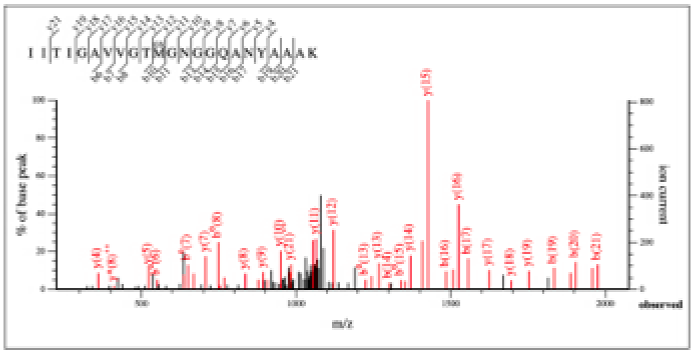

(B) Y151F

Peptide View

MS/MS Fragmentation of **IITIGSVVGTMGNGGQANFAAAK**
Found in **00004** in **Cronan_Zhe_FabG**, Hu Cronan EcFabG-Y mutant

Match to Query 10035: 2176.122648 from(1089.068600,2+) intensity(7158673.3000) scans(13497) rawscans(sn13497) rtinseconds(2286.1331) index(6526)
Title: 6527: Scan 13497 (rt=38.1022) [E:\Data\Fusion\Cronan\20-175-Cronan-Zhe-S-R.raw]
Data file E:\Data\Fusion\Cronan\20-175-Cronan-Zhe-S-R.raw

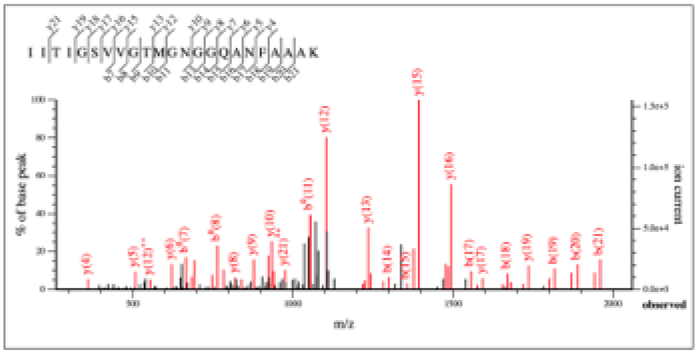

(C) K155A

Peptide View

MS/MS Fragmentation of **IITIGSVVGTMGNGGQANYAAAAAGLIGFSK**
Found in **00005** in **Cronan_Zhe_FabG**, Hu Cronan EcFabG-K mutant

Match to Query 14790: 2908.511412 from(970.511080,3+) intensity(15774782.0000) scans(17869) rawscans(sn17869) rtinseconds(2749.429) index(9495)
Title: 9496: Scan 17869 (rt=45.8238) [E:\Data\Fusion\Cronan\20-175-Cronan-Zhe-S-R.raw]
Data file E:\Data\Fusion\Cronan\20-175-Cronan-Zhe-S-R.raw

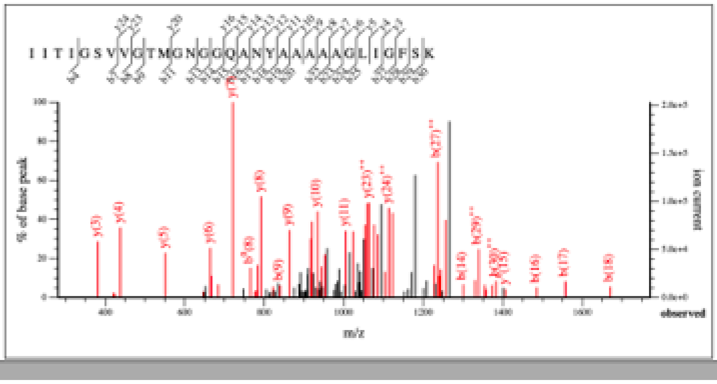

Fig. S5. Tandem MS spectra of the singly mutant FabG proteins. The spectra were chosen based on Mascot quality (136, 114 and 108 for panels A, B and C, respectively). and coverage from both the amino (*b* ions) and carboxyl (*y* ions) ends of the peptide. Panels A , B and C are the S138A, Y151F and K155A peptides, respectively. Note that MS/MS does not degrade sequentially from a given end but rather somewhat randomly such that *b* and *y* ions overlap. The fragments shown in red are those used in identification.

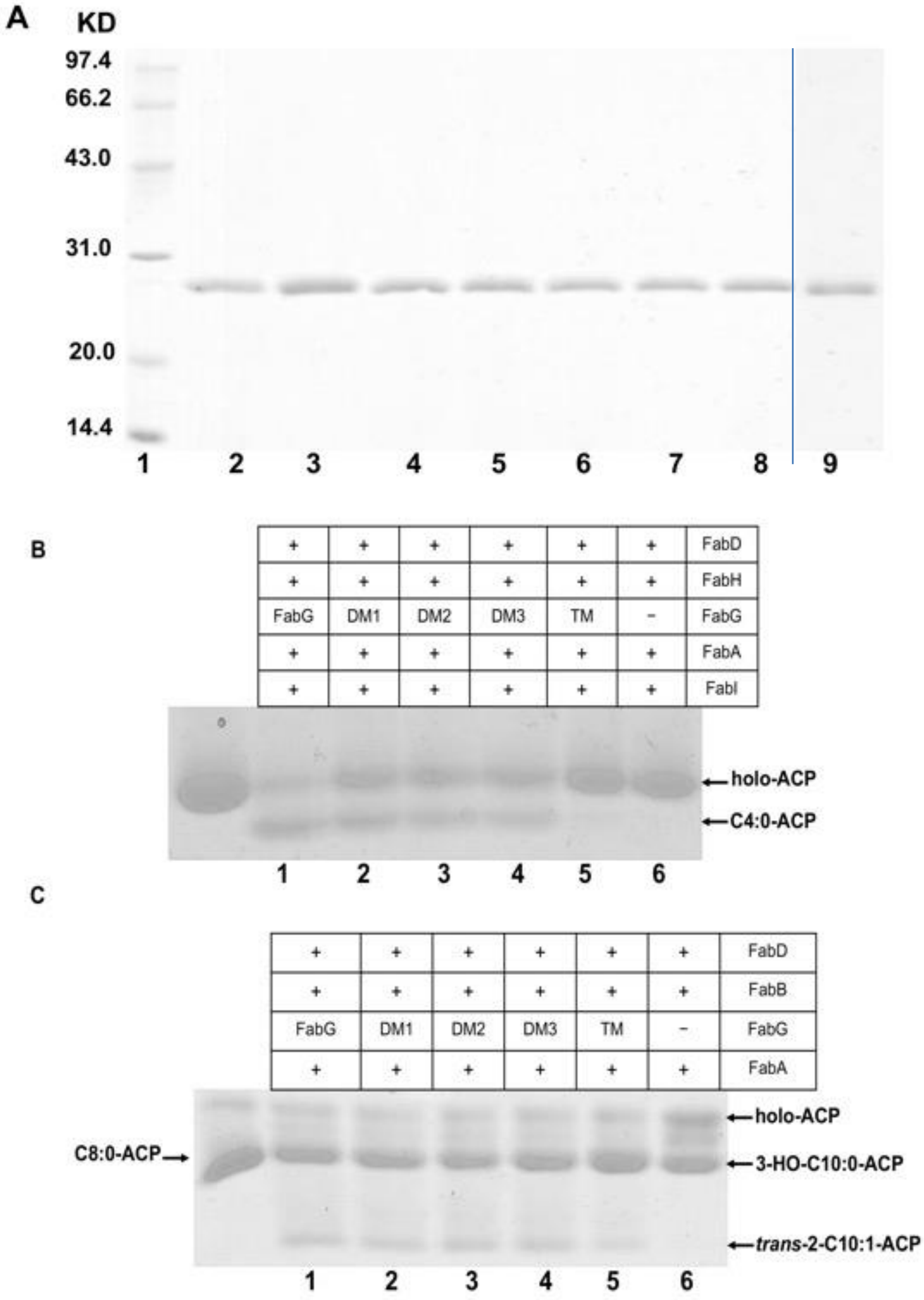
A
KD
97.4
66.2
43.0
31.0
20.0
14.4
1 2 3 4 5 6 7 8 9
B
+ + + + + + FabD
+ + + + + + FabH
FabG DM1 DM2 DM3 TM – FabG
+ + + + + + FabA
+ + + + + + FabI
holo-ACP
C4:0-ACP
1 2 3 4 5 6
C
+ + + + + + FabD
+ + + + + + FabB
FabG DM1 DM2 DM3 TM – FabG
+ + + + + + FabA
C8:0-ACP
holo-ACP
3-HO-C10:0-ACP
trans-2-C10:1-ACP
1 2 3 4 5 6

Fig. S6 Purification of FabG mutant proteins from *E. coli* strain BL21(DE3) and assay of FabG mutant protein function in fatty acid synthesis *in vitro*.

Panel A, purification of FabG and FabG mutant proteins by native nickel-chelate chromatography. Lane 1, molecular mass markers; lane 2, FabG S138A mutation protein; lane 3, FabG Y151F mutant protein; lane 4, FabG K155A mutation protein; lane 5, FabG S138A/Y151F mutant protein; lane 6, FabG S138A/K155A mutant protein; lane 7, Y151F/K155A mutant protein; lane 8, FabG S138A/Y151F/K155A mutant protein; lane 9, FabG wild type protein. Panel B, the initial cycle of fatty acid synthesis was reconstructed *in vitro* using a combination of FabH, FabG or FabG single mutation protein, FabA and FabI enzymes, NADH, and NADPH as cofactors, and malonyl-ACP plus acetyl-CoA as substrate to produce butyryl-ACP. Lane 1, wild type FabG (WT); lane 2, FabG S138A/Y151F (DM1); lane 3, FabG S138A/K155A (DM2); lane 4, FabG Y151F/K155A (DM3); lane 5, FabG S138A/Y151F/K155A(TM); lane 6, no addition of FabG protein. Panel C, the elongation reaction mixture contained FabB, FabG or FabG single mutation protein, FabA (EcFabA) enzyme, malonyl-ACP plus octanoyl-ACP as substrates, and NADH and NADPH as cofactors. Lane 1, wild type FabG (WT); lane 2, FabG S138A/Y151F; lane 3, FabG S138A/K155A (DM2); lane 4, FabG Y151F/K155A (DM3); lane 5, FabG S138A/Y151F/K155A(TM); lane 6, no addition of FabG protein. These proteins were purified from BL21(DE3) and purified by Ni chelate chromatography under denaturing conditions and refolded. Slot 9 was from a different gel.

A

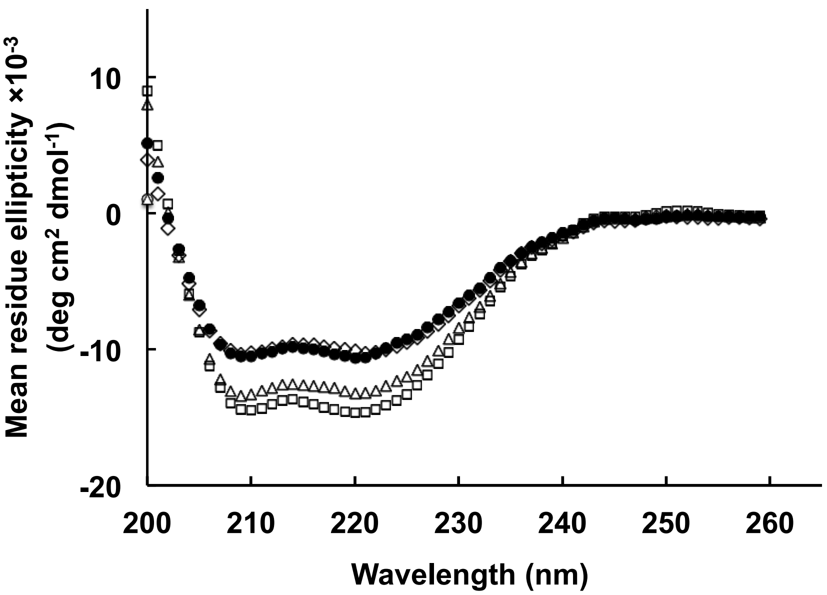

B

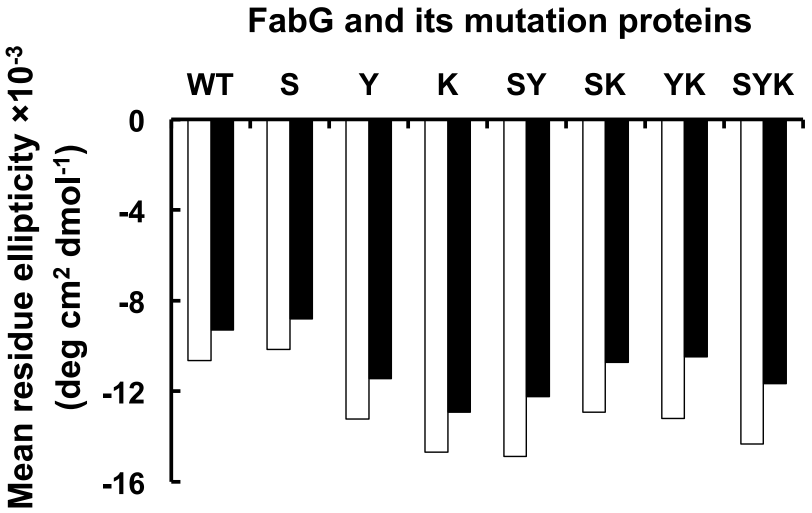

Fig. S7. Circular dichroism spectra of *E. coli* FabG and its mutation proteins and the effect of NADPH on the secondary structure of FabG and its mutation proteins. Panel A, circular dichroism spectra of *E. coli* FabG and its mutation proteins. CD spectra were measured at 25 °C. Filled cycle, wild type FabG; diamond, FabG S138A; triangle, FabG Y151F; square, FabG K155A. Panel B, Effect of NADPH on structure of mutant FabGs. White bar, no addition of NADPH; black bar, addition NADPH. WT, wild type

FabG; S, FabG S138A; Y, FabG Y151F; K, FabG K155A; SY, FabG S138A/Y151F; SK, FabG S138A/K155A, YK, FabG Y151F/K155A; SYK, FabG S138A/Y151F/K155A. These proteins were purified from BL21(DE3) and purified by Ni chelate chromatography under denaturing conditions and refolded.

References

[1] Lai C, Cronan JE. Isolation and characterization of beta-ketoacyl-acyl carrier protein reductase (*fabG*) mutants of *Escherichia coli* and *Salmonella enterica* serovar Typhimurium. J Bacteriol. 2004;186:1869-1878.

[2] Zhu L, Lin J, Ma J, Cronan JE, Wang H. Triclosan resistance of *Pseudomonas aeruginosa* PAO1 is due to FabV, a triclosan-resistant enoyl-acyl carrier protein reductase. Antimicrob Agents Chemother. 2010;54:689-698.

[3] Dennis JJ, Zylstra GJ. Improved antibiotic-resistance cassettes through restriction site elimination using Pfu DNA polymerase PCR. Biotechniques. 1998;25:772-776.

[4] Takeshita S, Sato M, Toba M, Masahashi W, Hashimoto-Gotoh T. High-copy-number and low-copy-number plasmid vectors for lacZ alpha-complementation and chloramphenicol- or kanamycin-resistance selection. Gene. 1987;61:63-74.
